# Prediction of axillary lymph node metastasis in breast cancer using preoperative CT Hounsfield units: A retrospective study

**DOI:** 10.1097/MD.0000000000050793

**Published:** 2026-09-25

**Authors:** Ayaka Koizumi, Hitoshi Sugimoto, Kenji Yamazaki, Akitoshi Nankaku, Jumpei Takashima, Daisuke Fujimoto, Fumihiko Miura, Hirotoshi Kobayashi

**Affiliations:** a Department of Surgery, Teikyo University School of Medicine, Mizonokuchi Hospital, Kawasaki-shi, Kanagawa, Japan.

**Keywords:** axillary lymph node metastasis, breast cancer, CT Hounsfield unit

## Abstract

Preoperative evaluation of axillary lymph node metastasis is crucial for selecting the optimal perioperative treatment for breast cancer. The most common methods for this evaluation include imaging tests, such as ultrasonography, and pathological assessments. This study investigated the utility of computed tomography (CT) Hounsfield units (HU) in diagnosing axillary lymph node metastasis. This retrospective study examined 218 patients with breast cancer who underwent surgery at our hospital between June 2017 and December 2023. We measured the HU of axillary lymph nodes using preoperative CT scans and defined the highest value as CT-HUmax. We explored the relationship between axillary lymph node CT-HUmax and the presence of metastasis. There were 56 patients in the axillary lymph node metastasis-positive group (P group) and 162 in the axillary lymph node metastasis-negative group (N group). The median CT-HUmax value was 48 (−25–69) for the P group and 30 (−17–73) for the N group, with the former having significantly higher values than those of the latter (*P* < .001). Similar findings were obtained with contrast-enhanced CT (CE-CT). Multivariate analysis revealed that CT-HUmax was a predictive factor for metastasis (odds ratio [OR]: 1.035; 95% confidence interval [CI]: 1.019–1.072; *P* = .011). The area under the receiver operating characteristic curve for CT-HUmax was 0.761 (95% CI: 0.688–0.834, *P* < .001). The cutoff value for CT-HUmax was 46, while the sensitivity, specificity, positive predictive value, negative predictive value, and accuracy rate using this value were 61%, 84%, 57%, 86%, and 78%, respectively. When CE-CT was used, the cutoff value was 134, and accuracy was 79%. CT-HU may be an effective predictor of axillary lymph node metastasis in breast cancer.

## 1. Introduction

In patients with breast cancer, the number of pathological regional lymph node metastases is a prognostic factor.^[1,2]^ Clinical-pathological characteristics – such as subtype, grade, and tumor size – as well as genomic analysis results influence the determination of treatment strategies for breast cancer. Among these factors, accurately assessing the status of lymph node metastasis is also critical information for selecting appropriate perioperative chemotherapy and postoperative radiotherapy.^[3–5]^ Preoperative chemotherapy is widely indicated for locally advanced and early stage breast cancer. Although there is no significant difference in overall survival or distant recurrence rates between postoperative and preoperative chemotherapy, a complete response is a favorable prognostic factor, highlighting the prognostic significance of preoperative chemotherapy.^[6,7]^ It is also advantageous for assessing drug sensitivity. Collectively, preoperative evaluation of lymph node metastases is conducive to optimal treatment selection.

Preoperative evaluation of axillary lymph node metastases is generally performed using various imaging techniques and pathological examination.^[8]^ Ultrasonography,^[9,10]^ contrast-enhanced magnetic resonance imaging (CE-MRI),^[8,11]^ and positron emission tomography (PET)^[12]^ are useful for predicting axillary lymph node metastases in breast cancer.

Contrast-enhanced computed tomography (CE-CT) is often performed for preoperative evaluation of metastasis in patients with Stage I or higher breast cancer and is commonly used for the evaluation of axillary lymph node metastasis following ultrasonography. Excluding cases that received neoadjuvant chemotherapy, computed tomography (CT) demonstrated a sensitivity of 78% and a specificity of 75% for detecting lymph node metastases with a short-axis diameter of 5 mm or greater. When morphological assessment was incorporated, the specificity for identifying metastases up to 5 mm increased to 90%, with an overall accuracy of 81%.^[13]^ In addition, the utility of the CT-Hounsfield unit (HU), rather than size or morphological assessment, in diagnosing axillary lymph node metastasis has also been suggested.^[14]^ However, few reports have examined this topic. In the present study, we investigated whether CT-HU is useful for diagnosing axillary lymph node metastasis in patients with breast cancer at our hospital.

## 2. Materials and methods

### 2.1. Patients

This retrospective study was approved by the ethics committee of our institution (approval no. 21-054; approval date: June 15, 2021) and adhered to the tenets of the Declaration of Helsinki and all institutional guidelines. We retrospectively extracted the information of patients who underwent breast cancer surgery at our hospital between June 2017 and December 2023. Consent was obtained using the opt-out method. A total of 318 patients underwent breast cancer surgery at our hospital during the study period. Among them, 100 were excluded for the following reasons: only the primary tumor was resected (n = 22), preoperative treatment was administered (n = 27), preoperative CT scans were not performed (n = 2), different CT devices were used (n = 47), or different imaging conditions were used (n = 2). Finally, 218 patients were included in this study (Fig. 1).

**Figure 1.**
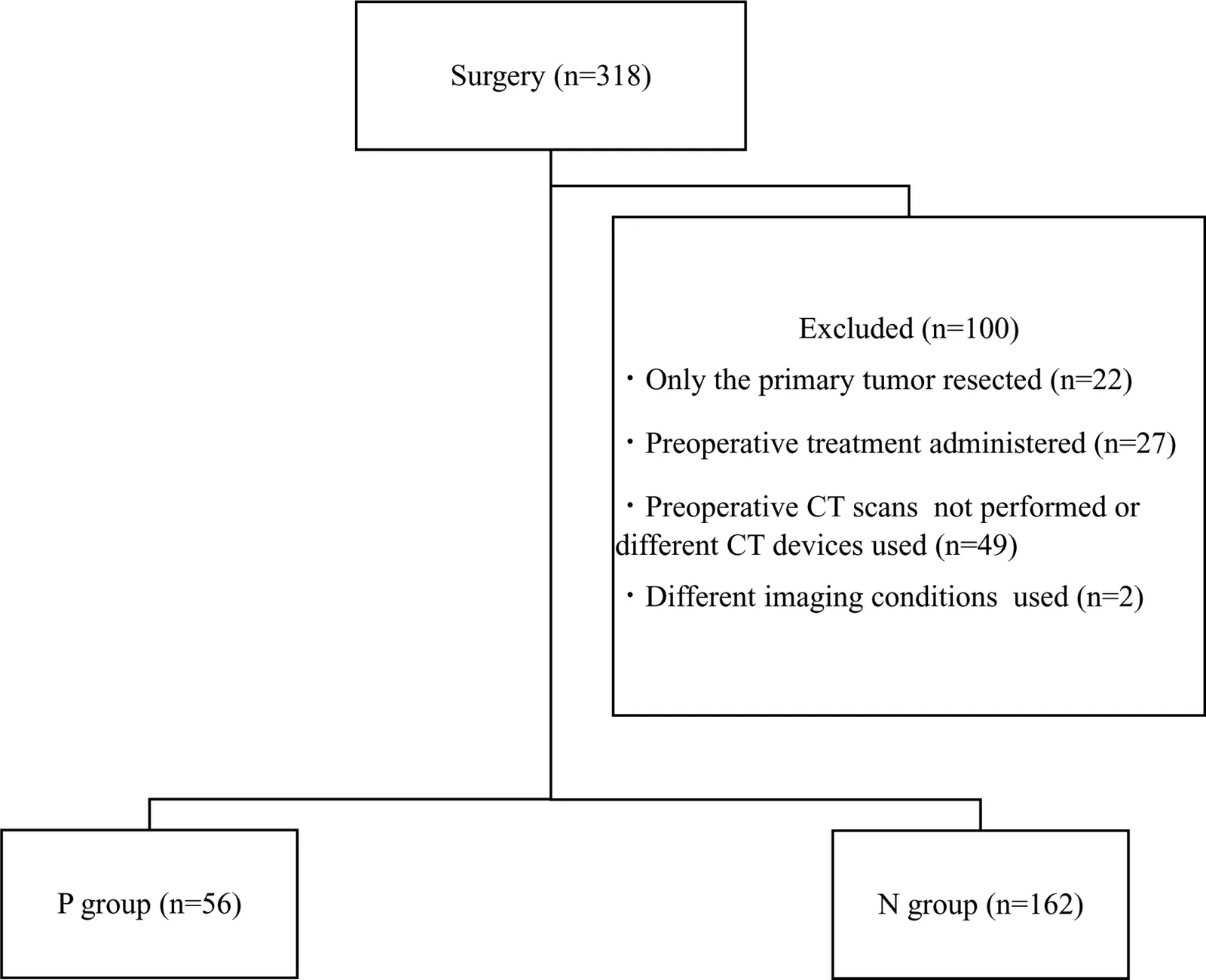
Patient selection flowchart. Of the 318 surgical patients, 100 were excluded, resulting in 218 included in the study: 56 patients in the axillary lymph node metastasis-positive group (P group) and 162 in the axillary lymph node metastasis-negative group (N group). CT = computed tomography.

### 2.2. CT examination

A multi-slice CT scanner (GE Healthcare, Little Chalfont, UK) was used. The following scanning parameters were used: tube voltage, 120 kVp; tube current, auto mA; rotation time, 0.4 seconds; reconstruction thickness, 5 mm; and detector configuration, 64 detector rows with a pitch of 1.375 mm/lot. Patients who had no contraindications for contrast agents underwent plain CT and CE-CT. Contrast agents containing 600 mg/kg of iodine were administered intravenously, and imaging scanning was started 75 seconds after the contrast flush. Patients who had a contraindication for contrast agents underwent only plain CT.

### 2.3. Evaluation of HU

In the CT scans obtained for preoperative evaluation, we evaluated the axillary lymph nodes on the affected side using plain CT-HU, CE-CT-HU, and CT-HU of aortography. For plain CT, 5 mm slices were used. For CE-CT, 0.625-, 1.25-, or 5-mm slices were used (a thin slice was selected from the above). The number of cases per slice was 7 for the 5-mm slice, 152 for the 1.25-mm slice, and 44 for the 0.625-mm slice. The axillary lymph nodes to be evaluated were defined as Level 1. For axillary lymph nodes on the affected side that could be identified, a region of interest (ROI) was placed on each slice, and the maximum HU was measured. Of the maximum HUs measured, the highest value was defined as the CT-HUmax. The ROI was delineated as an ellipse, ensuring that blood vessels, calcifications, and artifacts were avoided during its placement. Although the measurement range encompassed the fatty tissue surrounding the lymph nodes, this did not influence the maximum value. HU is a unit that represents the degree of X-ray absorption in CT images, with water assigned a value of 0 and air a value of −1000.^[15]^ Fat tissue has a lower value than water, while soft tissue has a higher value. Since lymph nodes – which are soft tissue – have a higher value than fat tissue, it is believed that the surrounding fat tissue does not affect the maximum value of the lymph nodes. Consequently, the lymph nodes were positioned to be fully contained within the ROI. The CT value of the aorta was measured by placing one ROI on the aortic arch, and the maximum value was defined as Ao max. The lymph aorta ratio (LAR) was defined as the CE-CT-HUmax/Ao max. All HU measurements were performed by the author.

### 2.4. Histopathological examination

All lymph nodes removed during axillary surgery were cut into individual blocks and stained with hematoxylin and eosin for pathological analysis. Both micro- and macro-metastases were defined as metastatic lymph nodes. The staging, histological type, and pathological grade of the primary tumor were classified according to the latest breast cancer treatment guidelines. Subtype classification and Ki-67 proliferative index were also examined using immunostaining.

### 2.5. Statistical analysis

For comparisons of age, pathological tumor factor, pathological grade, histological type, subtype, Ki-67, axillary surgery, plain CT-HUmax, CE-CT-HUmax, and LAR between cases of negative and positive axillary lymph node metastasis, Fisher exact test was used for categorical variables, and Mann–Whitney *U* test was used for continuous variables. Logistic regression analysis was performed, and covariates with *P* values < .05 in the univariate analysis were included in the multivariate analysis. The Youden index of the receiver operating characteristic (ROC) curve was used to set the cutoff values for CT-HUmax and CE-CT-HUmax. Using these cutoff values, we calculated the sensitivity, specificity, positive predictive value, negative predictive value, and accuracy. Statistical analyses were performed using SPSS for Mac, ver. 29.0.2.0 (IBM Corp., Armonk), and the open-source EZR software (version 1.52; The R Foundation for Statistical Computing, Vienna, Austria).

## 3. Results

### 3.1. Patient characteristics

Table 1 shows the clinical and pathological characteristics of all patients. The group with axillary lymph node metastasis was defined as Group P, and the group without axillary lymph node metastasis was defined as Group N. Group P included 56 patients (26%), and Group N included 162 patients (74%). The clinical and pathological characteristics of the groups were compared. The median age was 62 years (range, 34–93 years), with no significant difference between Group P (median age 61 years, 34–93 years) and Group N (median age 64 years, 34–88 years) (*P* = .93). Regarding axillary surgery, 79% of the patients underwent sentinel lymph node biopsy, and 21% underwent axillary lymph node dissection. In Group P, sentinel lymph node biopsy was performed in 11 patients (20%), and axillary lymph node dissection in 45 patients (80%). In Group N, sentinel lymph node biopsy was performed in 161 patients (99%), and axillary lymph node dissection in 1 patient (1%). A significant difference was observed between the 2 groups (*P* < .001). Regarding the pathological tumor factor, 17, 116, 72, 10, and 3 patients had pTis (8%), pT1 (53%), pT2 (33%), pT3 (5%), and pT4 status (1%), respectively, with a significant difference between the P and N groups (*P* < .001). No significant differences were observed between the 2 groups with regard to pathological grade, histological type, or subtype. The P group exhibited significantly higher Ki-67 expression than the N group (*P* = .03).

**Table 1:** Patients’ characteristics and comparison of groups.

Variables	All patients (N = 218)	Axillary lymph node		*P* value
		Group N* (n = 162)	Group P† (n = 56)	
Age (years), median (range)	62 (34–93)	64 (34–88)	61 (34–93)	.93
Pathological tumor factor				
Tis	17 (8%)	17 (11%)	0	<.001
T1	116 (53%)	94 (58%)	22 (39%)	
T2	72 (33%)	47 (29%)	25 (45%)	
T3	10 (5%)	2 (1%)	8 (14%)	
T4	3 (1%)	2 (1%)	1 (2%)	
Grade‡				
1	116 (56%)	90 (59%)	26 (46%)	.27
2	42 (20%)	29 (19%)	13 (23%)	
3	51 (24%)	34 (22%)	17 (30%)	
Unknown	9 (4%)	9 (6%)	0	
Histological type				
Ductal	177 (81%)	130 (80%)	47 (84%)	.14
Lobular	13 (6%)	11 (7%)	2 (4%)	
Mucinous	14 (6%)	13 (8%)	1 (2%)	
Others	14 (6%)	8 (5%)	6 (11%)	
Subtype				
Luminal	150 (69%)	116 (73%)	34 (61%)	.31
Luminal HER2	33 (15%)	21 (13%)	12 (21%)	
HER2	13 (6%)	10 (6%)	3 (5%)	
Triple negative	20 (9%)	13 (8%)	7 (13%)	
Unknown	2 (1%)	2 (1%)	0	
Ki-67 (%), range	10 (1–90)	10 (1–90)	10 (3–75)	.03
Axillary surgery				
SLNB	172 (79%)	161 (99%)	11 (20%)	<.001
ALND	46 (21%)	1 (1%)	45 (80%)	

ALND = axillary lymph node dissection; HER2, human epidermal growth factor receptor; SLNB = sentinel lymph node biopsy.

* Group N: group without axillary lymph node metastasis.

† (b) Group P: group with axillary lymph node metastasis.

‡ Grade: pathological grade.

### 3.2. Comparison of CT-HUmax, CE-CT-HUmax, and LAR between the groups

Table 2 shows the CT-HUmax, CE-CT-HUmax, and LAR values for the P and N groups. Of the 218 patients, 217 underwent plain CT. One patient underwent only a CE-CT scan. Although the reason why a plain CT scan was not performed for one patient is unclear, the attending physician did not order it for some reason. That patient was included in the N group. The plain CT analysis included 161 cases in the N group and 56 cases in the P group. The median CT-HUmax was 35 (−25–73), 48 (−25–69) in the P group and 30 (−17–73) in the N group, with a significantly higher value in the P group (*P* < .001). CE-CT was performed in 203 patients. Fifteen patients had conditions that precluded the use of contrast agents, such as a history of asthma or allergies; of these 15, 11 were in the N group and 4 were in the P group. The CE-CT-HUmax was 120 (8–209), 134 (80–209) in the P group, and 117 (8–164) in the N group, being significantly higher in the P group (*P* < .001). Similar results were obtained for LAR.

**Table 2:** Comparison of CT-HUmax, CE-CT-HUmax, and LAR between the groups.

	All patients (n = 217)	Axillary lymph node		*P* value
		Group N* (n = 161)	Group P† (n = 56)	
CT-HUmax, median (range)	35 (−25–73)	30 (−17–73)	48 (−25–69)	<.001

	All patients (n = 203)	Axillary lymph node		*P* value
		Group N* (n = 151)	Group P† (n = 52)	
CE-CT-HUmax	120 (8–209)	117 (8–164)	134 (80–209)	<.001
LAR	0.65 (0.045–1.13)	0.62 (0.045–0.82)	0.72 (0.39–1.13)	<.001

CE-CT = contrast-enhanced CT, CT = computed tomography, HU = Hounsfield unit, LAR = lymph aorta ratio.

* Group N: group without axillary lymph node metastasis.

† Group P: group with axillary lymph node metastasis.

### 3.3. Univariate and multivariate logistic regression analyses

Univariate and multivariate logistic regression analyses were performed for axillary lymph node metastases (Table 3). In the univariate analysis, the maximum diameter of the primary tumor (odds ratio [OR]: 1.041; *P* = .002), Ki-67 (OR: 1.016; *P* = .044), CT-HUmax (OR: 1.062; *P* < .001), and CE-CT-HUmax (OR: 1.052; *P* < .001) were associated with axillary lymph node metastasis. Multivariate analysis revealed that the maximum diameter of the primary tumor (OR: 1.045; *P* < .001), CT-HUmax (OR: 1.035; *P* = .011), and CE-CT-HUmax (OR: 1.041; *P* < .001) were significant predictive factors.

**Table 3:** Univariate and multivariate logistic regression analyses of multiparametric features associated with axillary lymph node metastasis.

Variables	Univariate analysis			Multivariate analysis		
	OR	95% CI	*P* value	OR	95% CI	*P* value
Age	1.016	0.984–1.05	.326			
Histological type Ductal or other	0.876	0.31–2.475	.802			
Maximal invasion diameter of primary region	1.041	1.015–1.067	.002	1.045	1.019–1.072	<.001
Grade* 1 or ≥2	1.95	0.796–4.777	.144			
Subtype Luminal or other	1.746	0.92–3.312	.088			
Ki-67	1.016	1–1.032	.044	1.003	0.984–1.023	.750
CT-HUmax	1.062	1.038–1.086	<.001	1.035	1.019–1.072	.011
CE-CT-HUmax	1.052	1.031–1.073	<.001	1.041	1.018–1.065	<.001

CE-CT = contrast-enhanced CT, CI = confidence interval, CT = computed tomography, HU = Hounsfield unit, OR = odds ratio.

* Grade: pathological grade.

### 3.4. Prediction of axillary lymph node metastasis using HU

The Youden index of the ROC curve was used to set the cutoff values for CT-HUmax and CE-CT-HUmax (Figs. 2 and 3). The area under the ROC curve was 0.761 (95% confidence interval [CI]: 0.688–0.834; *P* < .001) for CT-HUmax and 0.749 (95% CI: 0.665–0.832; *P* = .004) for CE-CT-HUmax. Among patients who underwent both plain CT and CE-CT scans, the areas under the ROC curve were 0.751 and 0.748, respectively, and no significant difference was observed (*P* = .936). The cutoff value for CT-HUmax was 46, while the sensitivity, specificity, positive predictive value, negative predictive value, and accuracy rate using this value were 61%, 84%, 57%, 86%, and 78%, respectively (Table 4). The cutoff value for CE-CT-HUmax was 134, with a sensitivity, specificity, positive predictive value, negative predictive value, and accuracy rate of 56%, 87%, 59%, 85%, and 79%, respectively.

**Table 4:** Diagnostic capability of CT-HU.

	Sensitivity	Specificity	Positive predictive value	Negative predictive value	Accuracy rate
CT-HUmax	61%	84%	57%	86%	78%
CE-CT-HUmax	56%	87%	59%	85%	79%

CE-CT = contrast-enhanced CT, CT = computed tomography, HU = Hounsfield unit.

**Figure 2.**
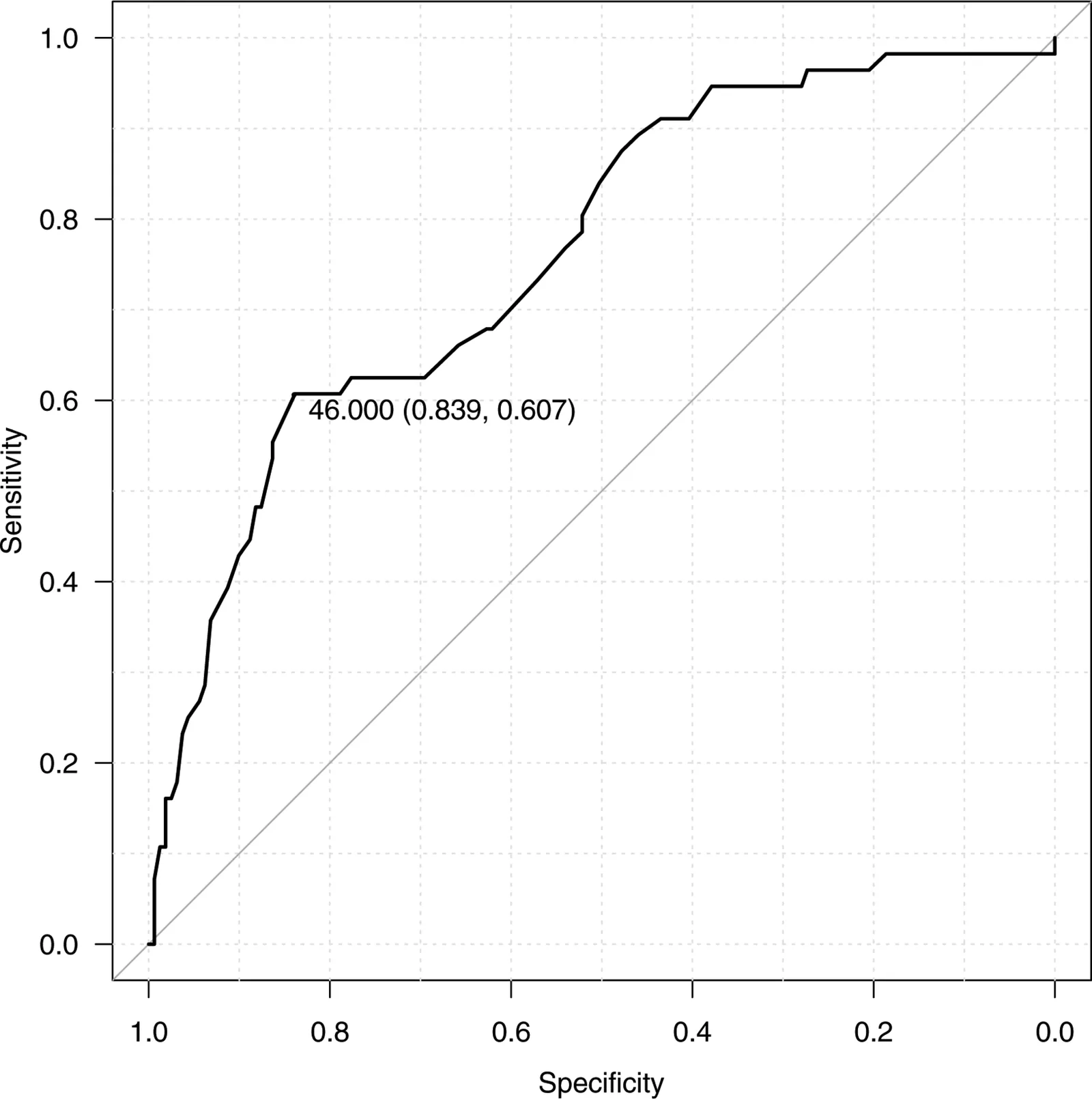
Youden index of the ROC curve for CT-HUmax. The cutoff value was 46, with an area under the ROC curve of 0.761 (95% CI: 0.688–0.834; *P* < .001). CI = confidence interval, CT = computed tomography, HU = Hounsfield Unit, ROC = receiver operating characteristic.

**Figure 3.**
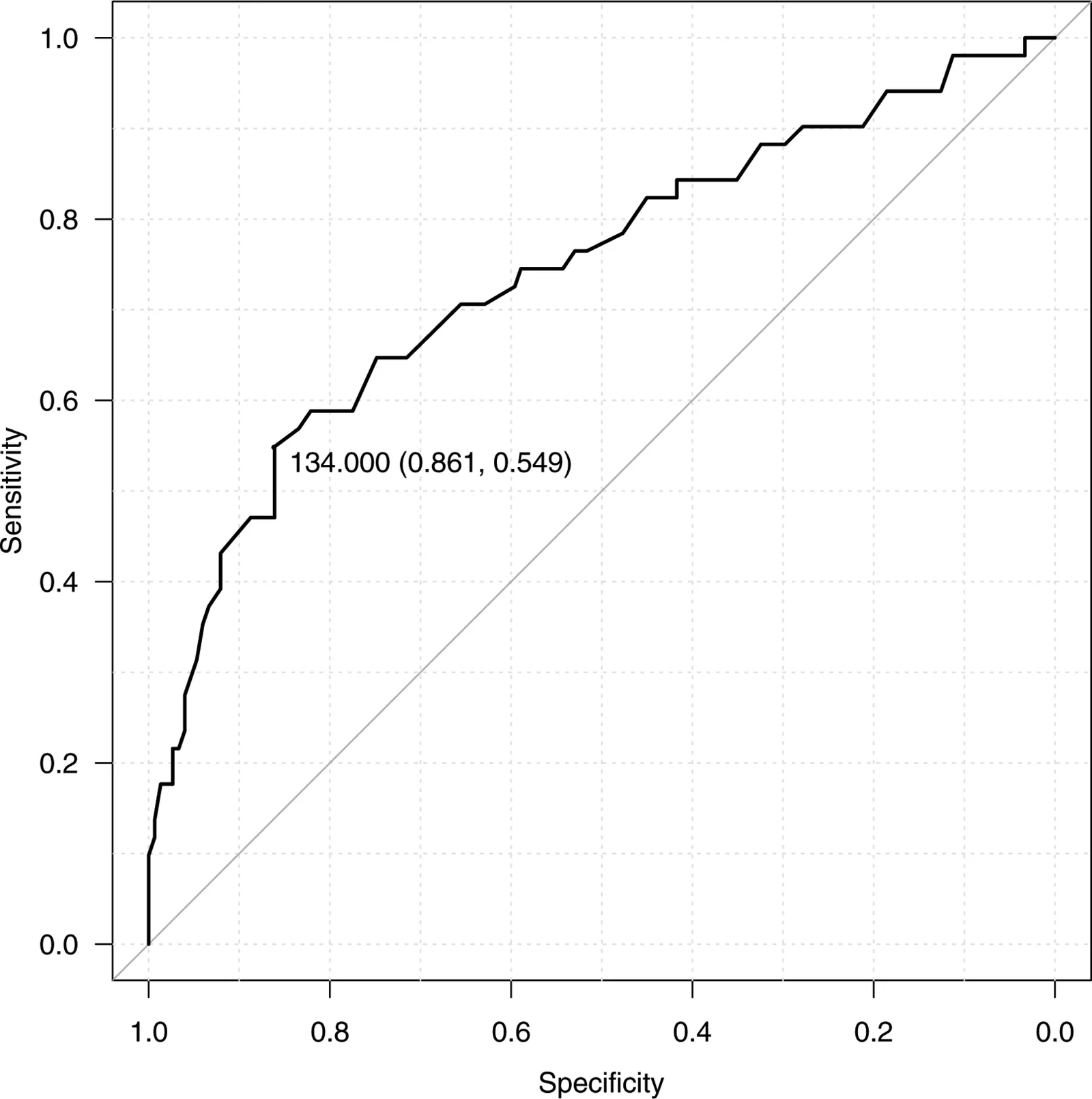
Youden index of the ROC curve for CE-CT-HUmax. The cutoff value was 134, with an area under the ROC curve of 0.749 (95% CI: 0.665–0.832; *P* = .004). CI = confidence interval, CE-CT = contrast-enhanced CT, CT = computed tomography, HU = Hounsfield unit, ROC = receiver operating characteristic.

## 4. Discussion

The present study demonstrated that HU is effective in the preoperative diagnosis of axillary lymph node metastasis in breast cancer. The sensitivity, specificity, positive predictive value, negative predictive value, and accuracy rate were 61%, 84%, 57%, 86%, and 78%, respectively, when using the CT-HUmax cutoff value. In a previous study, these values were 80%, 81%, 71%, 87%, and 80%, respectively.^[14]^ Although the sensitivity and positive predictive value in the present study were lower than those previously reported, both studies demonstrated a similar association between metastasis of axillary lymph nodes in breast cancer and CT-HU. Regarding sample size, both this study (n = 218) and the previous report (n = 131) had insufficient numbers, yet yielded similar results. Furthermore, considering that similar findings were obtained despite differences in CT equipment and imaging methods, similar findings are encouraging, but external validation is needed. We performed a multivariate analysis to confirm both CT-HUmax and CE-CT-HUmax as predictive factors for axillary lymph node metastasis. Furthermore, this study found no significant difference in diagnostic performance between plain-CT and CE-CT. However, because this was a retrospective study and slice thickness could not be standardized between plain CT and CE-CT, caution is required when interpreting these results. Plain CT was performed with 5-mm slice thickness in all cases. Evaluation of axillary lymph node metastasis using CT-HU and 5-mm slices and without using contrast media would reduce medical costs. Generally, preoperative CT is performed for Stage I or higher breast cancer. Since this test is routinely performed as a preoperative examination, axillary lymph node metastasis can be evaluated using CT-HU without the requirement for a new examination, thus being a cost-effective method. Furthermore, although morphological evaluation is subjective, CT-HU can be objectively evaluated as a continuous value. However, since CT-HU depends on the operator for ROI selection, lymph node selection, and slice thickness, it cannot be considered completely objective.

Regarding the preoperative evaluation of axillary lymph node metastasis, previous studies have reported the effectiveness of various imaging modalities. Ultrasonography is typically the first-line modality because it can be easily performed at the bedside. A previous systematic review reported that the sensitivity and specificity for diagnosing metastasis based on lymph node size ranged from 49 to 87% and 55 to 97%, respectively. For morphological evaluation, the sensitivity ranged from 26 to 76%, and specificity ranged from 88 to 98%.^[9]^ In a previous meta-analysis, the combination of ultrasonography and cytological or histological examination significantly improved the diagnostic accuracy for preoperative axillary lymph node metastasis, with a sensitivity of 80% and a specificity of 98%.^[10]^ It is clear that ultrasonography is the test with the highest diagnostic accuracy for evaluating axillary lymph nodes in breast cancer. However, despite being minimally invasive and easy to perform, the diagnostic performance of ultrasonography may vary depending on the operator’s skill and quality of the equipment. CE-MRI can also be useful in evaluating axillary lymph node metastases by extending the imaging field to include the axilla; previous studies have reported high specificity for this modality.^[8,11]^ A systematic review of 7 studies reported that PET had a sensitivity of 56% (95% CI: 44–67) and specificity of 96% (95% CI: 90–99) for diagnosing axillary lymph node metastases. However, its sensitivity was lower for small metastases, and only 11% (95% CI: 5–22) for micrometastases smaller than 2 mm.^[12]^

In the present study, we reported a sensitivity of 61%, specificity of 84%, and overall diagnostic accuracy of 78%. Although the specificity was high, the sensitivity was relatively low, showing a pattern similar to that of CE-MRI and PET. Therefore, integrating multiple parameters – such as CT attenuation values (CT-HU), lymph node size, and morphological characteristics – may improve the assessment of axillary lymph node metastasis using CT. Further research is needed.

Regarding the relationship between CT-HU and axillary lymph node metastasis, the reason behind the risk of metastasis increasing when the CT-HUmax is high remains unclear. CT-HU expresses the density of tissue as a relative value when water is defined as 0 and air as −1000.^[15]^ Therefore, in plain CT, higher CT-HU reflects increased tissue attenuation, possibly due to tumor cell infiltration.

CT-HU has also been applied in other medical fields. In many orthopedic studies, a strong correlation between bone mineral density and CT-HU has been established, leading to widespread exploration of opportunistic CT-HU screening for osteoporosis.^[16,17]^ In the field of otolaryngology, a strong inverse correlation has been reported between the histological grade of otosclerosis and the CT-HU value of the ROI, with some studies using CT-HU to evaluate the grade of otosclerosis.^[18]^ These studies have proposed that CT-HU indicates tissue hardness. In the present study, we found that metastatic lymph nodes with a high CT-HU value had increased tissue attenuation compared to lymph nodes without metastasis. Lymph nodes with metastasis demonstrate a pathological increase in tissue density in areas occupied by tumor cells, which can be detected in CT-HU images. Following this principle, lymph node metastases that appear unchanged in size or shape may still be identified preoperatively through CT-HU by evaluating tissue density. Regarding CE-CT, CT-HU may reflect high blood flow in the lymph nodes. Therefore, metastatic lymph nodes may have high blood flow and CT-HUmax values.

This study has some limitations. First, since this was a retrospective study from a single center, it may have been subject to various biases. Furthermore, this single-center study resulted in an insufficient sample size, necessitating the accumulation of more cases in the future. Second, given that the measurements were performed by the author alone and that the results were not blinded, the possibility of measurement bias cannot be ruled out. Furthermore, measurement bias may arise because the slice thickness used for plain CT and CE-CT was not standardized. While all plain CT scans used 5-mm slices, CE-CT scans included 7 cases with 5-mm slices, 152 cases with 1.25-mm slices, and 44 cases with 0.625-mm slices. Consequently, the comparisons of diagnostic performance between plain CT and CE-CT should be interpreted with caution. Third, while the N group primarily underwent sentinel lymph node biopsy, the majority of the P group underwent axillary lymph node dissection. Since axillary lymph node dissection involves examining a larger number of lymph nodes than sentinel lymph node biopsy, the probability of detecting metastasis is higher in patients who underwent axillary lymph node dissection. Therefore, the N group may include patients with undiagnosed metastases. Fourth, the cutoff values were unreliable because they may vary depending on the CT scanner used. Furthermore, since diagnostic performance was evaluated using the same dataset used to define the cutoff values, the diagnostic performance may have been overestimated. Studies involving prospective clinical trials are needed to elucidate the underlying factors in the correlation between a high CT-HU and the presence of axillary lymph node metastasis.

## 5. Conclusion

Preoperative CT-HU of axillary lymph nodes in breast cancer may be a predictor of axillary lymph node metastasis. Nevertheless, further research is needed to further examine and improve the diagnostic accuracy of this factor.

## Acknowledgments

The authors thank Editage (https://www.editage.com) for English language editing.

## Author contributions

**Conceptualization:** Hitoshi Sugimoto.

**Data curation:** Ayaka Koizumi.

**Formal analysis:** Ayaka Koizumi.

**Investigation:** Ayaka Koizumi, Akitoshi Nankaku.

**Methodology:** Hitoshi Sugimoto.

**Project administration:** Hirotoshi Kobayashi.

**Resources:** Ayaka Koizumi.

**Supervision:** Ayaka Koizumi.

**Validation:** Jumpei Takashima, Kenji Yamazaki.

**Visualization:** Ayaka Koizumi.

**Writing – original draft:** Ayaka Koizumi.

**Writing – review & editing:** Ayaka Koizumi, Hitoshi Sugimoto, Daisuke Fujimoto, Fumihiko Miura, Hirotoshi Kobayashi.

## References

[1] Beenken SW Urist MM Zhang Y. Axillary lymph node status, but not tumor size, predicts locoregional recurrence and overall survival after mastectomy for breast cancer. Ann Surg. 2003;237:732–8; discussion 738. 10.1097/01.SLA.0000065289.06765.71 PMC1514516 12724640

[2] Giuliano AE Connolly JL Edge SB. Breast Cancer-Major changes in the American Joint Committee on Cancer eight edition cancer staging manual. CA Cancer J Clin. 2017;67:290–303. 10.3322/caac.21393 28294295

[3] Toi M Imoto S Ishida T. Adjuvant S-1 plus endocrine therapy for oestrogen receptor-positive, HER2-negative, primary breast cancer: a multicentre, open-label, randomised, controlled, phase 3 trial. Lancet Oncol. 2021;22:74–84. 10.1016/S1470-2045(20)30534-9 33387497

[4] Rastogi P O’Shaughnessy J Martin M. Adjuvant abemaciclib plus endocrine therapy for hormone receptor-positive, human epidermal growth factor receptor 2-negative, high-risk early breast cancer: results from a preplanned monarchE overall survival interim analysis, including 5-year efficacy outcomes. J Clin Oncol. 2024;42:987–93. 10.1200/JCO.23.01994 PMC10950161 38194616

[5] Whelan TJ Olivotto IA Parulekar WR; MA.20 Study Investigators. Regional nodal irradiation in early-stage breast cancer. N Engl J Med. 2015;373:307–16. 10.1056/NEJMoa1415340 PMC4556358 26200977

[6] Early Breast Cancer Trialists’ Collaborative Group (EBCTCG). Long-term outcomes for neoadjuvant versus adjuvant chemotherapy in early breast cancer: meta-analysis of individual patient data from ten randomised trials. Lancet Oncol. 2018;19:27–39. 10.1016/S1470-2045(17)30777-5 PMC5757427 29242041

[7] Cortazar P Zhang L Untch M. Pathological complete response and long-term clinical benefit in breast cancer: the CTNeoBC pooled analysis. Lancet. 2014;384:164–72. 10.1016/S0140-6736(13)62422-8 24529560

[8] Chang JM Leung JWT Moy L Ha SM Moon WK. Axillary nodal evaluation in breast cancer: state of the art. Radiology. 2020;295:500–15. 10.1148/radiol.2020192534 32315268

[9] Alvarez S Añorbe E Alcorta P López F Alonso I Cortés J. Role of sonography in the diagnosis of axillary lymph node metastases in breast cancer: a systematic review. AJR Am J Roentgenol. 2006;186:1342–8. 10.2214/AJR.05.0936 16632729

[10] Houssami N Ciatto S Turner RM Cody HS 3rd Macaskill P. Preoperative ultrasound-guided needle biopsy of axillary nodes in invasive breast cancer: meta-analysis of its accuracy and utility in staging the axilla. Ann Surg. 2011;254:243–51. 10.1097/SLA.0b013e31821f1564 21597359

[11] Zhou P Wei Y Chen G Guo L Yan D Wang Y. Axillary lymph node metastasis detection by magnetic resonance imaging in patients with breast cancer: a meta-analysis. Thorac Cancer. 2018;9:989–96. 10.1111/1759-7714.12774 PMC6068453 29877048

[12] Cooper KL Meng Y Harnan S. Positron emission tomography (PET) and magnetic resonance imaging (MRI) for the assessment of axillary lymph node metastases in early breast cancer: systematic review and economic evaluation. Health Technol Assess. 2011;15:iii1–iv134. 10.3310/hta15040 PMC4781096 21276372

[13] Shien T Akashi-Tanaka S Yoshida M. Evaluation of axillary status in patients with breast cancer using thin-section CT. Int J Clin Oncol. 2008;13:314–9. 10.1007/s10147-007-0753-z 18704631

[14] Urata M Kijima Y Hirata M. Computed tomography Hounsfield units can predict breast cancer metastasis to axillary lymph nodes. BMC Cancer. 2014;14:730. 10.1186/1471-2407-14-730 PMC4193134 25266250

[15] DenOtter TD Schubert J. Hounsfield Unit. In: StatPearls. StatPearls Publishing; 2023. https://www.ncbi.nlm.nih.gov/books/NBK547721/. Accessed March 1, 2024. 31613501

[16] Lubbad O Hagos A Lubbad L El-Tahlawy Y Morassi GL Mazarakis NK. Diagnostic performance of lumbar computed tomography Hounsfield unit thresholds for osteoporosis and osteopenia: a systematic review and meta-analysis [published online ahead of print April 10, 2026]. Osteoporos Int. doi: 10.1007/s00198-026-07863-4. 10.1007/s00198-026-07863-4 41961088

[17] Courtois EC Ohnmeiss DD. Assessing bone quality in hounsfield units using computed tomography: what value should be used to classify bone as normal or osteoporotic? Eur Spine J. 2025;34:493–7. 10.1007/s00586-024-08565-3 39576307

[18] Quesnel AM Ishai R Meehan T. Computed tomography density as a bio-marker for histologic grade of otosclerosis: a human temporal bone pathology study. Otol Neurotol. 2022;43:e605–12. 10.1097/MAO.0000000000003535 PMC9245145 35761453

